# Antibacterial Activity of Ethanolic Extract of* Syzygium polyanthum* L.* (Salam)* Leaves against Foodborne Pathogens and Application as Food Sanitizer

**DOI:** 10.1155/2017/9024246

**Published:** 2017-12-19

**Authors:** Suzita Ramli, Son Radu, Khozirah Shaari, Yaya Rukayadi

**Affiliations:** ^1^Department of Food Science, Faculty of Food Science and Technology, Universiti Putra Malaysia (UPM), 43400 Serdang, Selangor, Malaysia; ^2^Laboratory of Natural Products, Institute of Bioscience, Universiti Putra Malaysia (UPM), 43400 Serdang, Selangor, Malaysia

## Abstract

The aim of this study was to determine antibacterial activity of* S. polyanthum* L.* (salam)* leaves extract foodborne pathogens. All the foodborne pathogens were inhibited after treating with extract in disk diffusion test with range 6.67 ± 0.58–9.67 ± 0.58 mm of inhibition zone. The range of MIC values was between 0.63 and 1.25 mg/mL whereas MBC values were in the range 0.63 mg/mL to 2.50 mg/mL. In time-kill curve,* L. monocytogenes* and* P. aeruginosa* were found completely killed after exposing to extract in 1 h incubation at 4x MIC. Four hours had been taken to completely kill* E. coli*,* S. aureus*,* V. cholerae,* and* V. parahaemolyticus* at 4x MIC. However, the population of* K. pneumoniae*,* P. mirabilis,* and* S. typhimurium* only reduced to 3 log CFU/mL. The treated cell showed cell rupture and leakage of the cell cytoplasm in SEM observation. The significant reduction of natural microflora in grapes fruit was started at 0.50% of extract at 5 min and this concentration also was parallel to sensory attributes acceptability where application of extract was accepted by the panellists until 5%. In conclusion,* S. polyanthum* extract exhibits antimicrobial activities and thus might be developed as natural sanitizer for washing raw food materials.

## 1. Introduction

Food safety is a major concern for both consumers and food manufacturers alike. Despite the high degree of awareness of food preservation methods, the occurrence of disease outbreaks caused by foodborne pathogens and spoilage microorganisms in foods is still increasing [1]. Foodborne illness is also known as foodborne disease and colloquially referred to food poisoning is any illness resulting from the consumption of contaminated food, pathogenic bacteria, viruses, or parasites that contaminate food, rather than chemical or natural toxins. The symptoms for food poisoning are including diarrhea, fever, vomiting, abdominal pain, and dehydration [2]. Currently to preserve food from spoilage, some manufacturers used synthetic antimicrobial agents to prevent the growth of food spoilage and food pathogenic microorganisms include benzoates, nitrates, and nitrites [3]. However, emergence of microbial resistance to classic antimicrobial agents becomes a major health concern due to elevated use of chemical preservatives in food processing [4]. Nowadays, consumers are more aware on food safety especially on the long term effect of synthetic additives in food including toxic and carcinogenic effect. Hence, this issue has led to the increased demand for high-quality, minimally processed foods with extended shelf-life and preferably free from or with a low level of synthetic additives in food [5]. Moreover, foods need to be safe and fresh with prolonged shelf-life. Therefore, antimicrobials agent from natural plants is a good source as an alternative to synthetic preservatives in order to satisfy consumers demand for safe and healthy food [6]. Antimicrobial agents can be either synthesized or naturally occurring in plant materials [7]. The main reasons for adding antimicrobial in food are to control food spoilage and to prevent the growth of foodborne pathogens [8]. This suggests that natural plants might be sources of antimicrobials agents that can be used to inhibit the growth of foodborne pathogens.

*S. polyanthum* L., which is synonym to* salam*, is a deciduous tropical tree belonging to the Myrtaceae family [9]. This plant grows wildly on lowlands and is widely distributed in the temperate, subtropical, and tropical regions in the world [10]. These leaves had several name based on the location including* S. polyanthum* in Malaysia and Indonesia it is called* serai kayu* (Malay);* meselangan* is the name that used in Sumatra,* gowok* (Sunda),* salam* (Java, Sunda, Madura),* manting *(Java), or* kastolam* (Kangean) [11].* S. polyanthum* leaves have been used traditionally as medicine or therapeutic agents including efficiency against ulcer, hypertension, diabetes, hyperuricemia, diarrheal, gastritis, skin diseases, and inflammation [11, 12]. Furthermore,* S. polyanthum* leaves were believed to possess antibacterial activity against* Streptococcus mutans* [11] and* Staphylococcus aureus* [13]. Besides that, this plant also had antifungal activities against spoilage fungi* Euroticum* spp.,* Aspergillus* spp., and* Penicillium* spp. [14]. Furthermore, according to Perumal et al. [10],* S. polyanthum* leaves are also found to be noncytotoxic to normal mammalian cell lines. Based on previous study,* S. polyanthum* leaves had antibacterial activity against* B. cereus* and* B. subtilis* [15].

Therefore the aim of this study was to determine the antimicrobial activity of* S. polyanthum* leaves extracts against a wide spectrum of foodborne pathogens.

## 2. Materials and Methods

### 2.1. Samples

Dried* S. polyanthum* leaves were purchased from Herbal Market Bandung, Indonesia, deposited, and identified in Institute of Bioscience (IBS), Universiti Putra Malaysia.

### 2.2. Preparation of Extract

One hundred grams of dried* S. polyanthum *leaves was ground using dry blender. Then, the samples were soaked in 400 mL absolute ethanol for seven days at room temperature as stated by Rukayadi et al. [16], with some modification. The mixture was then filtered using Whatman number 2 filter paper and concentrated by using rotary evaporator at 50°C and at speed of 150 rpm for 60 to 90 min. The extract was dissolved in 10% dimethylsulfoxide (DMSO) to obtain stock solution. The final concentration of extract was standardized at 10 mg/mL or 1%. The stock solution was kept at −4°C.

### 2.3. Bacteria Cultures

A total of nine strains of frequently reported as foodborne pathogens were included:* Escherichia coli* O157:H7 ATCC 43895,* Klebsiella pneumoniae* ATCC 13773,* Listeria monocytogenes* ATCC 19112,* Proteus mirabilis* ATCC 21100,* Pseudomonas aeruginosa* ATCC 9027,* Salmonella typhimurium* ATCC 14028,* Staphylococcus aureus *ATCC 29737,* Vibrio cholerae* (Isolate 2), and* Vibrio parahaemolyticus* ATCC 1780. All the microbial strains used in this study were maintained by subculturing them on the nutrient agar (NA) or nutrient agar mix with 3% of NaCl for* V. cholerae* and* V. parahaemolyticus* and were incubated overnight. Bacteria strains can be stored in this way for a few weeks on the agar plates before subculturing them again, while, for the stock culture preparation, 0.5 mL of overnight culture with broth media was mixed into 0.5 mL of 80% sterile glycerol. Cultures were stored at −20°C. These stock cultures were kept from 6 months to 1 year [17].

### 2.4. Disk Diffusion Test

*S. polyanthum* extract was tested for antimicrobial activity using the disk diffusion method as described by CLSI [18]. Bacteria species with concentration in range 10^6^–10^8^ CFU/mL were spread on Mueller Hinton agar (MHA) with a sterile cotton swab. Sterile filter paper discs with 6 mm diameter were placed on top of the culture and 10 *μ*L of 10 mg/mL (w/v) of* S. polyanthum* leaves extract was loaded on the paper discs. 0.1% of commercial chlorhexidine (CHX) was used as positive control whereas 10% DMSO as negative control. The plates were incubated at 37°C for 24 hours. Evidence of clear zone indicates bacterial growth inhibition and the diameter was measured in mm.

### 2.5. Determination of Minimum Inhibitory Concentration (MIC) and Minimum Bactericidal Concentration (MBC)

Determination of MIC and MBC values was performed using a method described in the CLSI [18]. MIC was conducted in 96-well U-shaped microtiter plate using twofold standard broth microdilution method with an inoculum of approximately 10^6^–10^8^ CFU/mL.* S. polyanthum* leaves extract with concentration 10 mg/mL was mixed and twofold diluted in the respective medium containing inoculum. Column 12 of the microtiter plate contained the highest concentration of extract (5 mg/mL) while column 3 contained the lowest concentration of extract (0.0097 mg/mL). Column 1 served as negative control (only medium, no inoculum, and no antimicrobial agent), while column 2 served as positive control for all samples (only medium and inoculum or antimicrobial agent-free well) for 24 hours. The MIC was defined as the lowest concentration of antimicrobial agent that was able to inhibit the visible growth [16] while minimal bactericidal concentration (MBC) was standing for the lowest concentration of antimicrobial agent that completely killed the growth of culture. MBC was determined by subculturing the suspension (10 *μ*L) from each well in microtiter plate on MHA. The plates were then incubated at 37°C for 24 hours or until growth was seen at positive control.

### 2.6. Time-Kill Curve Assay

A time-kill curve assay was carried out with the MIC values found previously in the microplate bioassay, using a modification of the viable cells count method of de Souza et al. [19].* S. polyanthum* leaves extract was diluted with the Muller Hinton broth (MHB) medium containing inoculum of approximately 10^6^–10^8^ CFU/mL to obtain final concentrations of 0x MIC, 0.5x MIC, 1x MIC, 2x MIC, and 4x MIC for each bacterial species. At different time intervals of exposure, (0, 0.5, 1, 2, and 4 hours), 0.1 mL of the suspension was serially diluted in 1% phosphate buffered saline (PBS) and plated onto MHA. The plates were incubated at 37°C for 24 hours. The results were expressed in log CFU/mL.

### 2.7. Scanning Electron Microscope (SEM)

Fresh* K. pneumoniae* and* S. aureus* culture was treated with the extract and incubated at 37°C in MHB for 24 hours. The pellets were collected by centrifugation (5000 ×g for 10 min) and were fixed with 2.5% glutaraldehyde for 4–6 hours at 4°C. Then, the pellets were washed with 0.1 M sodium cacodylate buffer for 10 min and were repeated for 3 times. The pellets were then postfixed with 1% osmium tetroxide for 2 hours at 4°C, washed again with 0.1 M sodium cacodylate buffer for 10 min, and repeated for 3 times. Then the pellets were dehydrated using 35, 50, 75, and 95% acetone for 15 min each. Lastly the pellets were dehydrated using 100% acetone for 15 min and were repeated for 3 times. Cell suspensions were transferred into a specimen basket, made from aluminium foil coated with albumin, and then put in critical dryer for 0.5 hours. The specimens were mounted on a stub and the sputter was coated with gold. The morphology of the cells was observed and images were obtained using SEM instrument.

### 2.8. Application of* S. polyanthum* Extract as Food Sanitizer on Grapes

The samples of grapes fruit (approximately 10 g) were treated with tap water and natural sanitizer with concentration of 0.05%, 0.50%, 1.00%, and 5.00% of* S. polyanthum* extract according to Yusoff et al. [20] with slight modification. Grapes fruit was soaked separately at different time interval, 5, 10, and 15 min, to determine their microflora growth viability. Untreated samples remained unwashed. For bacteria growth determination, 1 mL from each treatment was diluted into 10^−1^, 10^−2^, and 10^−3^ dilution. Then, 0.1 mL from each dilutions series was spread on the different types of agar, Plate count agar, Eosin Methylene Blue agar (EMB), and Baird Parker agar, and incubated at 37°C for 24 hours. The presence of colonies was counted.

### 2.9. Evaluation of Sensory Attributes Acceptability of Treated Grapes Fruit

The sensory evaluation acceptability test was performed according to Brasil et al. [21], with slight modification. A group of 50 untrained panellists were presented with five different 3-digit coded samples placed in a random order. The evaluation was conducted based on the 9-point hedonic scale for inspection acceptance testing where panellists assessed each treated sample in terms of colour (observed with eyes), odour (smelled with nose), and the texture (touched with finger). The ratings for the each analysis of samples were given in a scale ranging from extremely disliked (scale of 1) to extremely liked (scale of 9).

## 3. Results

### 3.1. Yield of Extract

100 g of dried weight of* S. polyanthum* leaves was extracted using ethanol solvent and yielded 8.21 g of extract, which gave the percentage value of 8.21% total yield.

### 3.2. Disk Diffusion Test

The inhibition zone of* S. polyanthum* leaves extract against foodborne pathogens is shown in Table 1. The inhibition zones were between 6.67 ± 0.58 and 9.67 ± 0.58 mm. Results showed the inhibition zones of* S. polyanthum* extract were 7.00 ± 0.28 mm, 9.33 ± 0.50 mm, 9.67 ± 0.58 mm, 7.00 ± 0.32 mm, 6.67 ± 0.58 mm, 9.33 ± 0.58 mm, 6.67 ± 0.50 mm, 8.33 ± 0.58 mm, and 6.67 ± 0.58 mm on* E. coli, K. pneumoniae, L. monocytogenes, P. aeruginosa, P. mirabilis, S. aureus, S. typhimurium, V. cholerae,* and* V. parahaemolyticus,* respectively. The larger inhibition zone gave the meaning of higher antibacterial activity of the extract on the tested microbial species.

### 3.3. Determination of Minimum Inhibitory Concentration (MIC) and Minimum Bactericidal Concentration (MBC)

From the result shown in Table 2,* S. polyanthum *leaves extract demonstrated broad-spectrum activity against all selected bacteria with the MIC values ranging from 0.63 to 1.25 mg/mL. Among them* L. monocytogenes* and* S. aureus* were found to be the most susceptible pathogens with the MIC value of 0.63 mg/mL. Results show that the MBC was in the range of 0.63 mg/mL to 2.50 mg/mL.* L. monocytogenes *gave the lower MBC value compared to other strains which was 0.63 mg/mL.

### 3.4. Time-Kill Curve Assay

**Figure 1:**
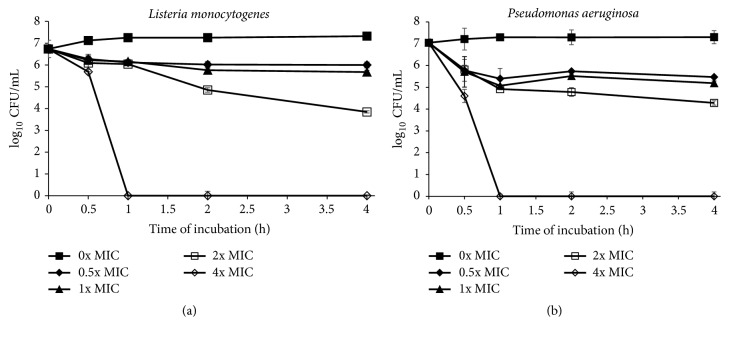
(a) Time-kill curve plots for* L. monocytogenes* (0, 0.315, 0.630, 1.260, and 2.520 mg/mL) following exposure to* S. polyanthum* L. extract. Values given in the brackets after species are 0x MIC, 0.5x MIC, 1x MIC, 2x MIC, and 4x MIC, respectively. (b) Time-kill curve plots for* P. aeruginosa* (0, 0.625, 1.250, 2.500, and 5.000 mg/mL) following exposure to* S. polyanthum* L. extract. Values given in the brackets after species are 0x MIC, 0.5x MIC, 1x MIC, 2x MIC, and 4x MIC, respectively.

**Figure 2:**
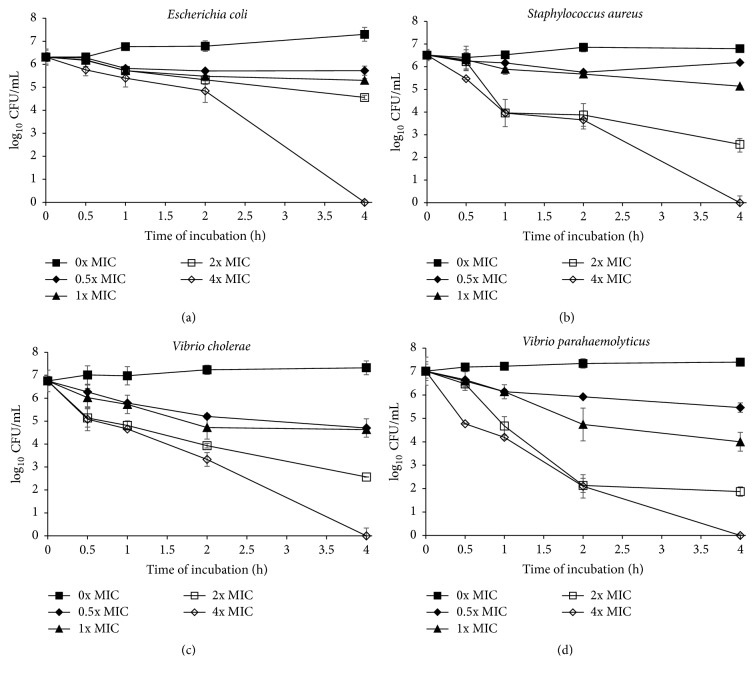
(a) Time-kill curve plots for* E. coli* O157:H7 (0, 0.625, 1.250, 2.500, and 5.000 mg/mL) following exposure to S*. polyanthum* L. extract. Values given in the brackets after species are 0x MIC, 0.5x MIC, 1x MIC, 2x MIC, and 4x MIC, respectively. (b) Time-kill curve plots for* S. aureus* (0, 0.315, 0.630, 1.260, and 2.520 mg/mL) following exposure to* S. polyanthum* L. extract. Values given in the brackets after species are 0x MIC, 0.5x MIC, 1x MIC, 2x MIC, and 4x MIC, respectively. (c) Time-kill curve plots for* V. cholerae* (0, 0.625, 1.250, 2.500, and 5.000 mg/mL) following exposure to* S. polyanthum* L. extract. Values given in the brackets after species are 0x MIC, 0.5x MIC, 1x MIC, 2x MIC, and 4x MIC, respectively. (d) Time-kill curve plots for* V. parahaemolyticus* (0, 0.625, 1.250, 2.500, and 5.000 mg/mL) following exposure to* S. polyanthum* L. extract. Values given in the brackets after species are 0x MIC, 0.5x MIC, 1x MIC, 2x MIC, and 4x MIC, respectively.

**Figure 3:**
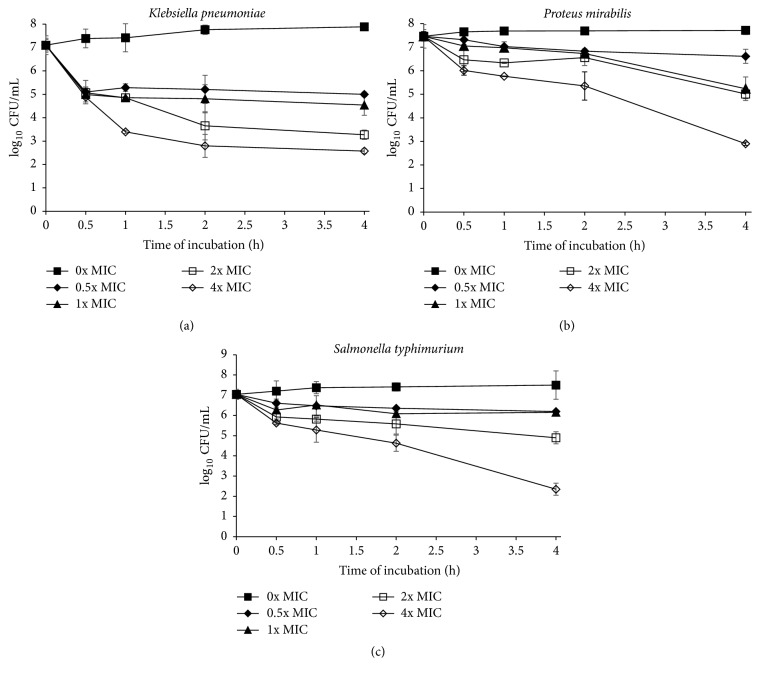
(a) Time-kill curve plots for* K. pneumoniae* (0, 0.625, 1.250, 2.500, and 5.000 mg/mL) following exposure to* S. polyanthum* L. extract. Values given in the brackets after species are 0x MIC, 0.5x MIC, 1x MIC, 2x MIC, and 4x MIC, respectively. (b) Time-kill curve plots for* P. mirabilis* (0, 0.625, 1.250, 2.500, and 5.000 mg/mL) following exposure to* S. polyanthum* L. extract. Values given in the brackets after species are 0x MIC, 0.5x MIC, 1x MIC, 2x MIC, and 4x MIC, respectively. (c) Time-kill curve plots for* S. typhimurium* (0, 0.625, 1.250, 2.500, and 5.000 mg/mL) following exposure to* S. polyanthum* L. extract. Values given in the brackets after species are 0x MIC, 0.5x MIC, 1x MIC, 2x MIC, and 4x MIC, respectively.

In this study, time-killing assay was done to find the correlation between the concentrations of* S. polyanthum* leaves extract with its killing effects on selected foodborne pathogens. Time-kill curve assay showed that* S. polyanthum* leaves extract can kill* L. monocytogenes* and* P. aeruginosa* at 4x MIC for 1 hour (Figures 1(a) and 1(b)) and* E. coli, S. aureus, V. cholerae,* and* V. parahaemolyticus* at 4x MIC for 4 hours (Figures 2(a), 2(b), 2(c), and 2(d)). The population of* K. pneumoniae*,* P. mirabilis*, and* S. typhimurium* also showed a reduction < 3 log_10_ CFU/mL when treated with the extract at 4x MIC for 4 hours as shown in Figures 3(a), 3(b), and 3(c).

### 3.5. Scanning Electron Microscope (SEM)

**Figure 4:**
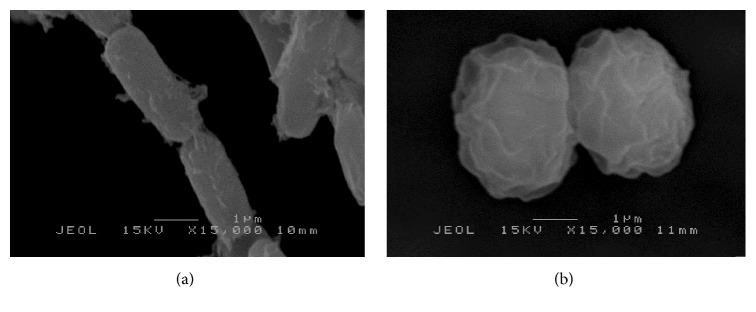
(a) Scanning electron micrograph of untreated* K. pneumoniae*. (b) Scanning electron micrograph of* K. pneumoniae *after treating with* S. polyanthum* L. extract at MIC value for 24 hours.

**Figure 5:**
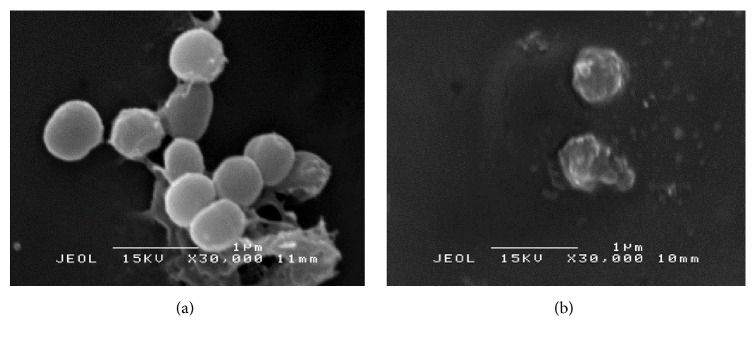
(a) Scanning electron micrograph of untreated* S. aureus*. (b) Scanning electron micrograph of* S. aureus* after treating with* S. polyanthum* L. extract at MIC value for 24 hours.

Figures 4(a) and 4(b) show the treated and untreated* K. pneumoniae* cells with* S. polyanthum* extract at the concentration of 1.25 mg/mL for overnight. The untreated* K. pneumoniae* showed normal cells characteristics with rod shape and intact peptidoglycan layer. Meanwhile, after treating with* S. polyanthum* extract overnight, cells appeared to be damaged with some irregularities surfaces, whereby the rod-shaped cells shrank and deflated, and some of them were cavitated. Besides that, the effect of* S. polyanthum* extract against* S. aureus* is shown in Figures 5(a) and 5(b). The grape-like cluster morphology of* S. aureus* was altered after the treatment. Disruptions with release of intracellular material associated with* S. aureus* cells losing their cytoplasm (empty and flaccid cells) were also observed.

### 3.6. Application of* S. polyanthum* Extract as Food Sanitizer on Grapes

Table 3 shows the effect of* S. polyanthum* against natural flora in grapes. Bacterial population which was detected in grapes includes* E. coli* and* S. aureus*. This study showed that total plate count had been reduced significantly after exposure to 0.50% at 5 min soaking where the population decreased from 5.78 ± 0.05 to 5.19 ± 0.13 log_10_ CFU/mL. On the other hand,* E. coli*'s population only had been significantly reduced after treating at 1.00% for 5 min and decreased to undetected at 5% extract at 5 min treatment while* S. aureus* decreased to log_10_  0.00 ± 0.00 CFU/mL starting at 0.50% in 5 min.

### 3.7. Evaluation of Sensory Attributes Acceptability of Treated Grapes Fruit

Table 4 shows the sensory acceptability of treated grapes with* S. polyanthum* extract. Based on the result, it can be concluded that most panellists accepted these grapes samples which were washed with extracts and tap water with overall acceptability of more than scale 7. There is also no significant difference between washing treatment using highest concentrations of extract (5%) and tap water. That means that panellist is not able to differentiate between using tap water and extracts. Therefore,* S. polyanthum* did not affect the physical appearances of grapes.

## 4. Discussion

A recent trend in food processing is to avoid the use of chemical preservatives. Thus, natural antimicrobial alternatives are required. In this research, ethanol was used as a solvent. Ethanol is also classified as a polar solvent. This means that this solvent is miscible in water and it will extract mostly the ionic compounds from* S. polyanthum* leaves. Ethanol has better dissolving capabilities compared to water because it has a slightly low dipole and is dielectric; thus it is slightly polar [22]. Moreover, according to Marriott [23], the solvents permitted for use in the preparation of food ingredients are ethanol, ethyl acetate, and acetone only.

From the disk diffusion result,* L. monocytogenes* gave the highest inhibition zone compared to others strain. On the other hand,* P. mirabilis*,* S. typhimurium,* and* V. parahaemolyticus* were observed to be more resistant against the extract. Generally, in Gram-negative bacteria, their outer membranes serve as permeability barrier which allows only small hydrophilic molecules to pass through into the cell, restricting their rate of penetration for certain antimicrobial compounds and excluding larger molecules. Besides, they also possess multidrug resistant pumps which exclude some of the antibacterial compounds across the barrier [24]. These special buildings make the Gram-negative bacteria more tolerant to any foreign compounds intake. On the other hand, disk diffusion test sometimes gave inaccurate result due to some limitations such as the ability of extract to pass through the pore discs and the inability of hydrophobic compounds to diffuse into the media agar [25]. In addition according to Gangoué-Piéboji et al. [26], by using disc, some active compounds might be blocked in the disc pores and are unable to pass through the inoculated media and hence cannot express their activity. Besides that, inhibition zone of 0.1% of CHX against the pathogens was in range of 8.80 ± 0.58 to 12.00 ± 0.00 mm. This finding showed lower inhibition zone compared to the study done by Abbas et al. [27], which mentions that the inhibition zone was between 13.84 ± 0.65 and 14.87 ± 0.53 mm on* E. faecalis* by using 2% of CHX. This observation may be due to the different concentration of CHX. However, according to Gupta et al. [28], inhibition zone of CHX against* P. aeruginosa* was 10.00 mm, whereas* S. aureus* was 11.00 mm. Therefore, the finding was similar to this present study. In conclusion, the disc-diffusion test is normally used as first screening in the detection of active compounds in plant extracts before further determination was performed.

*L. monocytogenes* and* S. aureus *were found to be the most susceptible pathogen with the MIC value of 0.63 mg/mL while the other strains showed 1.25 mg/mL.* L. monocytogenes* also showed the lower MBC values compared to other strains with 0.63 mg/mL. Besides that,* S. typhimurium, V. cholerae,* and* V. parahaemolyticus* had the same value for MIC and MBC, meaning this bacteria can be inhibited and killed at the same concentration of plant extract. This result showed that Gram-positive bacteria were easier to inhibit compared to Gram-negative ones. Gram-negative bacteria have a hydrophilic outer membrane rich in lipopolysaccharide molecules. Therefore it serves as a penetration barrier towards macromolecules [29]. Although this description is widely accepted, and accepted for many essential oils, some researchers have stated that the Gram distinction may have little relation to growth inhibition and some herbs are equally effective against both groups of bacteria [30]. However, the outer membrane is not completely impermeable as there are porin proteins present in this layer that can create channels large enough to allow restricted passage of molecules with a molecular mass below 600 Da, such as substituted phenolics in herb extracts and essential oils, allowing their slow penetration into the periplasmic space and the cytoplasmic membrane [31]. Thus it is possible that over a longer contact time the active compounds present in leaves extract would have the same effect on Gram-negative and Gram-positive bacteria [32]. Besides that, the* Euphorbia hirta* extract showed lower antimicrobial activity on* E. coli* compared to* S. polyanthum* extract with the MIC value of 3.13 mg/mL [33]. According to Rand et al. [34],* S. polyanthum* extract demonstrated better bactericidal and bacteriostatic properties compared to* B. oleracea* extract where MIC and MBC value of* S. aureus*,* E. coli*,* P. aeruginosa,* and* K. pneumoniae* were 100 mg/mL and 400 mg/mL, 300 mg/mL and 400 mg/mL, 100 mg/mL and 200 mg/mL, and 100 mg/mL and 400 mg/mL, respectively.* Moringa oleifera *seed extract displayed weaker antibacterial activity compared to* S. polyanthum *with MIC values >4 mg/mL on* E. coli*,* P. aeruginosa,* and* S. typhimurium* [35]. Moreover,* S. polyanthum* also shows good antibacterial effect compared to garlic and ginger extract. Based on Smith-Palmer et al. [36], MIC and MBC of garlic and ginger extract on* L. monocytogenes*,* E. coli* and* S. aureus* were >1% whereas* S. polyanthum* gave bacteriostatic and bactericidal effect between 0.063% and 0.125% against the same bacteria strains. On the other hand,* S. polyanthum* and* Syzygium aromaticum* (clove) showed quite similar antibacterial activity. Inhibition zones of* S. aromaticum* against* E. coli*,* L. monocytogenes,* and* S. aureus* were 9.7, 8.4, and 8.0 mm, respectively. Meanwhile the MIC values were 0.04%, 0.03%, and 0.04% on the same pathogens. Besides that,* Syzygium cumini* showed no antimicrobial activity against* E. coli* and* K. pneumoniae;* however, it is effective against* S. aureus* with 9.00 mm shown in inhibition zone [37]. Therefore,* S. cumini* had lower antibacterial activity compared to* S. polyanthum* extract in terms of disk diffusion test. According to Chikowe et al. [38],* Syzygium forte*,* Syzygium francisii*,* Syzygium moorei*,* Syzygium puberulum,* and* S. wilsonii* illustrated weaker antibacterial activity compared to* S. polyanthum* where there was no inhibition zone against* P. mirabilis* and* S. aureus*. However,* S. francisii*,* S. moorei,* and* S. wilsonii* showed higher inhibition zone against* E. coli* compared to* S. polyanthum* extract. On the other hand, all the tested* Syzygium* spp. gave lower antibacterial activity against* E. coli* compared to* S. polyanthum* in terms of MIC value except* S. francisii* with 0.256 mg/mL. Apart from that,* S. polyanthum* had higher antibacterial activity against* K. pneumoniae* than* S. forte*,* S. francisii*,* S. moorei*,* S. puberulum,* and* S. wilsonii.*

Generally, different crude extracts show different antibacterial level among different microbes tested. These inconsistencies might be due to the different expression of the bioactive compounds present in the extracts. As suggested by Cowan [39], essential oils and polyphenolic compounds exhibited different bacteriostatic and bactericidal effect on bacterial strains. Therefore, minimum inhibitory concentration (MIC) is the parameter commonly used to guide the selection on the antimicrobial agent used in treatment by predicting their efficacy at a standard inoculum approximately 10^6^ CFU/mL after an incubation period of 18–24 hours [18]. However, MIC only provides limited information on the kinetics of the antimicrobial action. Due to this limitation, time-killing assay was performed in order to find the correlation between the rate of bactericidal activity with the incubation time and concentration of antimicrobial agent [32].

Figures 1(a) and 1(b) showed that both* L. monocytogenes* and* P. aeruginosa* had been completely killed at 4x MIC in 1-hour incubation with 2.52 and 5.00 mg/mL of* S. polyanthum *leaves extract, respectively. These two strains were killed earlier compared to others. However finding by Penduka and Okoh [40] stated that* L. monocytogenes* can be killed completely with 0.314 mg/mL of crude* Garcinia kola* seed methanol extract in 0.5 hours of incubation. Therefore this finding suggested that* S. polyanthum* leaves extract might possess lower antibacterial activity against* L. monocytogenes*. In other case, according to Alwash et al. [41],* Melastoma malabathricum *extract had been reported to be able to kill completely* P. aeruginosa* at concentration 1.56 mg/mL within 8 hours. The comparison was hard to evaluate as both extracts are effective in completely killing* P. aeruginosa* at different concentration and incubation time. Generally, more concentrated extract will be able to kill bacteria in short period.

*E. coli*,* S. aureus*,* V. cholerae,* and* V. parahaemolyticus* had been killed at 4x MIC within 4 hours as shown in Figures 2(a), 2(b), 2(c), and 2(d). Five mg/mL extract had been used to kill* E. coli* completely in 4 hours. In contrast, Mamman et al. [42], had reported the bactericidal activity of* Azadirachta indica* extract on* E. coli* strain was at concentration 250 mg/mL. Therefore, results from this study revealed that* S. polyanthum* leaves extract is a good antibacterial source against* E. coli* strain. According to Witkowska et al. [29], the bactericidal effect of sage extract on* S. aureus *was at concentration > 40 mg/mL for 24 hours of incubation time. In addition, rosemary and clove extracts were able to kill* S. aureus* completely at 5 and 10 mg/mL concentration for 4 and 6 hours of incubation time, respectively. However, from this finding,* S. polyanthum* leaves extract only took 4-hour incubation to kill* S. aureus* completely at concentration 2.52 mg/mL. In comparison,* S. polyanthum* leaves have better bacterial effect against* S. aureus* compared to sage, rosemary, and clove extract. Furthermore,* S. aureus* was Gram-positive bacteria where the membrane structure was easier to disrupt compared to Gram-negative bacteria. From finding by Kwieciński et al. [43], it stated that* S. aureus* can be killed within 15 min with 1% (v/v) of tea tree oil while El-Farmawi et al. [44] showed that methicillin-resistant* S. aureus* can be killed during 2–4 hours of incubation with cinnamon and green tea extract at concentration 300 *μ*l/mL and 200 *μ*l/mL, respectively. In conclusion,* S. polyanthum* leaves extract had a weaker bactericidal effect as compared to tea tree oil, cinnamon, and green tea extracts. The time-kill plot obtained for* V. cholerae* and* V. parahaemolyticus* strains exhibited bactericidal end points which were at 4x MIC after 4-hour incubation. However, the population of both pathogens was reduced approximately to 3 log at 2x MIC after 4 hours. Penduka and Okoh [40] reported that 69% of* V. parahaemolyticus* was killed at 5 mg/mL after 2-hour incubation using* Garcia kola* seed methanol extract. Therefore,* S. polyanthum* leaves have a quite similar bactericidal effect with* G. kola* seed extract where >50%* V. cholerae *and* V. parahaemolyticus*' population were completely killed at same concentration and incubation time.

On the other hand, the populations of* K. pneumoniae*,* P. mirabilis,* and* S. typhimurium* were only reduced to <3 log after 4-hour incubation as shown in Figures 3(a) and 3(b). According to Supardy et al. [45], extract able to reduce bacterial cell less than 3 log was indicated as having bacteriostatic effect. Furthermore, 3 log was the minimum level of microbial population to cause infection in human. According to El-Farmawi et al. [44],* K. pneumoniae* can be killed within 6 to 8 hours of incubation with cinnamon and green tea extract at concentrations 500 *μ*l/mL and 300 *μ*l/mL, respectively. Its means* S. polyanthum* leaves had a quite weaker antibacterial activity compared to cinnamon. Research by Rajeh et al. [46] reported the bactericidal activity of* Euphorbia hirta* extract on* P. mirabilis* was at concentration 50 mg/mL at 24-hour incubation. Muniandy et al. [47] stated that concentration 1.08 mg/mL of* Coleus aromaticus* extract can completely kill* P. mirabilis* within 24 hours of incubation time. On the other hand, Konaté et al. [48], reported the bactericidal effect of* Sida alba* extract on* P. mirabilis* at concentration 0.05 mg/mL within 6 hours of incubation. Results revealed that* S. polyanthum *leaves possess better antibacterial agent compared to* E. hirta* and* C. aromaticus *extracts; however this leaves extract showed lower bactericidal effect compared to* S. alba. *Foster [49] stated that* Salmonella* spp. had the ability to adapt in wide range of conditions including ability to grow in various pH and temperatures. Besides that, Mandal et al. [50] reported the reduction of* Salmonella *spp. until 2.19 log at concentration 0.512 mg/mL of* Camelia sinensis* extract within 24 hours of incubation time. Similarly, in this research,* Salmonella* spp. only reduced to 3 log and did not completely get killed after treatment with* S. polyanthum* leaves extract in 4-hour incubation. This showed that* S. polyanthum* had better antibacterial activity against* Salmonella* spp. compared to* C. sinensis* where the population reduction took only about 4 hours.

Increasing of plant extract's concentration will lead to diffusion into membrane cell thus causing membrane destruction [51]. In addition, the killing activity of* S. polyanthum* leaves extract was concentration-dependent. According to Miksusanti et al. [52], at higher concentration of extract, the membrane becomes leaky to cytoplasmic components which lead to cell death. It was also speculated that high concentrations of* S. polyanthum* leaves extract contribute to rapid killing of the microorganism because of the serious loss of membrane integrity and degenerative cell wall. In order to kill the microorganisms, leaves extract needs to bind, occupy, and remain at the target site for sufficient period of time to prevent the metabolic process and interfere with the chemical reactions of the bacteria. In addition, the increasing of plant extract can saturate the target site and cause rapid bactericidal effect [53]. The hydrophobicity of plants extract and their bioactive compounds contribute in the breaking down of the membrane cells lipid and make them more permeable for the penetration [54]. Furthermore, the bioactive compounds in extract may inhibit the synthesis of essential metabolites (folic acid) by preventing the enzymatic reaction. The protein synthesis in the microorganisms also can be inhibited if the bioactive compounds interfere and change the shape of ribosome. The interference can lead to misreading of the genetic code on the mRNA [55].

Action modes of extract against tested strains were observed as shown in Figures 4 and 5. The treated* K. pneumoniae* showed that the cell was ruptured and shrunk. This observation was supported by dos Santos et al. [56], where the electron-dense particles which stayed packed in cytoplasm before were dispersed and result in an empty hollow in cytoplasm. This indicated that cytoplasm's compartment was released acrossed the cell wall. Study conducted by Supardy et al. [45] also reported the damaged and distorted* K. pneumoniae* cell after treating with 0.5 mg/mL of* Halimeda discoidea* extract for 12, 24, and 36 hours of treatment. The same result also were obtained by Rajeshwari et al. [57] where the morphology of* K. pneumoniae* showed unusual shapes of expanding, swelling, shrinking, and other multiple disorientations that were absent in the control sets after treating with* H. discoidea* extract. In addition, the same phenomenon was reported by Derakhshan et al. [58] who treated the* K. pneumoniae* with the cumin (*Cuminum cyminum* L.) herb extract. However, not all shrunk cells after treatment represent cells death. Some of them decrease their cell surface area as the way of adaptation, in order to minimize the target site for antimicrobial compounds to attach on them [45]. However, constant exposure of plant extract with increasing of concentration and extended time treatment will eventually kill the cells [29]. Generally, the results showed the shrinkage and deformation of the cells proved that the cells were under a suppressive and stressful environment. From the results, the prompt antibacterial action on the cells was seen to specifically attack the cell membrane components. Moreover, the treated cell of* S. aureus* also showed morphology changes. The grape-like cluster morphology of* S. aureus *was altered after the treatment. Disruptions with release of intracellular material associated with* S. aureus* cells losing their cytoplasm (empty and flaccid cells) were also observed. The distortion of the physical structure of the cell could cause the expansion and destabilization of the membrane and increase membrane fluidity, which in turn increases the passive permeability and manifests itself as a leakage of various vital intracellular constituents, such as ions, ATP, nucleic acids, sugars, enzymes, and amino acids. This observation suggested that the ionic interactions between the cationic polymers and negatively charged lipopolysaccharides (as lipoteichoic acid, a component of the thick peptidoglycan layer of Gram-positive bacteria) in the outer membrane can be responsible for the growth inhibition and lysis, through blockage of important nutrients flow such as Ca^+2^ and Mg^+2^ ion entering the cell [59].

Fresh food including fruit and vegetables may harbour a variety of microbes which priory originating from the environment where they grew. The microbes will keep growing along the postharvest handling and food processing and caused spoilage to the foods if no proper decontamination methods applied [60]. The growing and survival of these microbes with prolonged time especially during storage period will spoil the foods and cause foodborne illness when consumed by people outside. As reported by Chang and Fang [61], the survivability of* E. coli* O157:H7 and* S. typhimurium* in shredded lettuce within 10–12 days imposed a potential health risk to consumers. In this study, treatment with tap water is referring to the common washing methods applied by household. There were some researchers who reported the capability of tap water to reduce the total bacterial count around 2 to 3 log_10_ CFU/mL [62, 63]. However, in this study, the treatment with tap water only showed slight reduction compared to previous study. Brackett [64] had reported that the use of tap water for washing cannot completely remove the bacterial populations on food materials. Besides, there are limitations of using tap water in washing food materials which is due to the presence of chlorine residues in treated tap water. Chlorine residues have become a concern in food safety due to their potentiality to produce carcinogenic compounds such as trihalomethanes, haloacetic acids, haloketones, and chloropicrin when reacting with organic matter [65]. As stated by Gill and Badoni [66], reusing of processing water as sanitizer will make the tap water another source of cross-contamination. In this study, the bacterial reduction in treated grapes fruit was proportional with the increasing of* S. polyanthum* extract concentration and soaking time. Research was in the similar of Abadias et al. [67] who also reported the reductions of microbial populations were increased as the concentration of sanitizer and washing time increased. However, study conducted by Tornuk et al. [68] proved that the ability of thyme sanitizer was affected by extract concentration while different exposure time did not give significant reduction on the bacterial populations in apple fruits. Therefore, the relative influence in terms of microbial inactivation was tap water < 0.05% < 0.50% < 1.00 < 5.00%.

As stated by Vilgis [69], the ideal sanitizer is when the panellists are unable to recognize the difference between treated and nontreated samples which gives the meaning of not much change occurring before or after the treatment was applied. Study reported by Kumudavally et al. [70] reported the effectiveness of clove extract on reducing the pathogenic microflora in fresh mutton until 4-day treatment at 25 ± 2°C, at the same time giving no adverse effect on physical and sensory qualities. In correlation with that, Solomon et al. [71] had reported the organoleptic and chemical evaluations of suya (boneless meat pieces) after treating with basil extract for 30, 60, 90, and 120 mins. In their sensory analysis part, authors reported that the suya soaked with basil extracts enhances eating quality as it improved the flavour of meat. However, most of the panellists were not satisfied in terms of the final colour of treated suya (brownish green colour). In this study, grapes were accepted by panellist even after treating with highest concentration of extract (5%). From this observation it can be concluded that, generally, the treated samples which had been exposed to highest concentration and longest exposure time were accepted by panellist. That means* S. polyanthum* extract did not affect or change the physical characteristics of food samples after exposure to highest concentration of extract at maximum time of exposure.

Antimicrobial activity of herbs and spices varies widely, depending on the several factors including spices type, test medium, and types of pathogens. Moreover, microorganisms differ in their resistance to different types of spices and herbs. According to Kalemba and Kunicka [72], active components of herbs at low concentrations may interact synergistically with other factors including sodium chloride, acids, and preservatives to increase preservation. However, antimicrobial activity of herb derived has been reported to diminish during food processing [73]. Therefore, further studies on the efficacy of these natural antimicrobial agents in a range of food products as well as evaluation of potential interactions of antimicrobial compounds with components of food matrices such as fats, carbohydrates, and proteins are required.

## 5. Conclusion

In conclusion, susceptibility test is very important step in the screening of antibacterial activity of plant material. From the result,* S. polyanthum* leaves had antibacterial activity against wide spectrum of foodborne pathogens and are able to reduce microflora count in fresh fruits. Therefore the plant might be promoted to further tests towards its evaluation as a sanitizer or preservative in wide range of foods.

## Figures and Tables

**Table 1:** Inhibition zone of *S. polyanthum *L. leaves extract against foodborne pathogens.

Strains	Inhibition zone (mm)		
	*S. polyanthum* extract	CHX	DMSO
*E. coli *O157:H7	7.00 ± 0.28	9.00 ± 0.00	n.a
*K. pneumoniae*	9.33 ± 0.50	11.50 ± 0.50	n.a
*L. monocytogenes*	9.67 ± 0.58	12.00 ± 0.00	n.a
*P. aeruginosa*	7.00 ± 0.32	10.00 ± 0.51	n.a
*P. mirabilis*	6.67 ± 0.40	10.00 ± 0.70	n.a
*S. aureus*	9.33 ± 0.52	10.00 ± 0.23	n.a
*S. typhimurium*	6.67 ± 0.50	8.00 ± 0.00	n.a
*V. cholerae*	8.33 ± 0.30	8.80 ± 0.58	n.a
*V. parahaemolyticus*	6.67 ± 0.50	9.00 ± 0.00	n.a

n.a: no activity; diameter of inhibition zones in mm (including disc); positive control (chlorhexidine: CHX; 0.1%); negative control (DMSO; 10%); results were expressed as means ± standard deviation (SD); *n* = 3 × 3.

**Table 2:** Minimum inhibitory concentration (MIC) and minimum bactericidal concentration (MBC) of *S. polyanthum *L. extract against foodborne pathogens.

Strains	MIC (mg/mL)	MBC (mg/mL)
*E. coli *O157:H7	1.25	2.50
*K. pneumoniae *	1.25	2.50
*L. monocytogenes *	0.63	0.63
*P. aeruginosa*	1.25	2.50
*P. mirabilis *	1.25	2.50
*S. aureus *	0.63	1.25
*S. typhimurium *	1.25	1.25
*V. cholerae*	1.25	1.25
*V. parahaemolyticus*	1.25	1.25

**Table 3:** Effect of different concentrations and exposure times of *S. polyanthum* L. extract on natural microbial in grapes.

Sample	Grapes								
Bacterial species	TPC (log_10_ CFU/mL)			*E. coli *(log_10_ CFU/mL)			*S. aureus *(log_10_ CFU/mL)		
ET/Treatment	5 min	10 min	15 min	5 min	10 min	15 min	5 min	10 min	15 min
Control	5.78 ± 0.05^aA^	5.78 ± 0.05^aA^	5.78 ± 0.05^aA^	4.14 ± 0.05^aA^	4.14 ± 0.05^aA^	4.14 ± 0.05^aA^	3.45 ± 0.03^aA^	3.45 ± 0.03^aA^	3.45 ± 0.03^aA^
Tap water	5.73 ± 0.08^aA^	5.72 ± 0.03^aA^	5.66 ± 0.11^aA^	4.11 ± 0.05^aA^	4.09 ± 0.03^aA^	4.05 ± 0.04^aA^	3.37 ± 0.11^aA^	3.14 ± 0.07^aA^	3.52 ± 0.06^aB^
0.05%	5.69 ± 0.04^aA^	5.39 ± 0.03^aA^	5.62 ± 0.16^aA^	4.08 ± 0.06^aA^	4.18 ± 0.03^aA^	4.10 ± 0.02^aA^	3.19 ± 0.07^bA^	3.33 ± 0.18^bA^	3.25 ± 0.14^bA^
0.50%	5.19 ± 0.13^bA^	5.16 ± 0.14^bA^	5.07 ± 0.03^bA^	3.88 ± 0.18^aA^	4.01 ± 0.04^aA^	3.95 ± 0.06^aA^	0.00 ± 0.00^cA^	0.00 ± 0.00^cA^	0.00 ± 0.00^cA^
1.00%	4.46 ± 0.22^cA^	4.39 ± 0.12^cAB^	3.03 ± 0.07^cB^	3.22 ± 0.06^bA^	3.46 ± 0.19^bAB^	3.11 ± 0.07^bB^	0.00 ± 0.00^cA^	0.00 ± 0.00^cA^	0.00 ± 0.00^cA^
5.00%	3.74 ± 0.03^dA^	3.49 ± 0.23^dA^	0.00 ± 0.00^dA^	0.00 ± 0.00^cA^	0.00 ± 0.00^cA^	0.00 ± 0.00^cA^	0.00 ± 0.00^cA^	0.00 ± 0.00^cA^	0.00 ± 0.00^cA^

Values with different small letters within the same columns are significantly different (*p* < 0.05). Values with different capital letters within the same rows are significantly different (*p* < 0.05). ET: Exposure Time.

**Table 4:** Sensory attributes acceptability of treated grapes with *S. polyanthum *L. extract.

Attributes	Tap water	0.05%	0.50%	1.00%	5.00%
Colour	8.49 ± 0.88^a^	8.16 ± 1.12^a^	8.10 ± 1.08^a^	7.65 ± 0.80^a^	7.81 ± 1.88^a^
Odour	8.59 ± 1.65^a^	7.11 ± 1.11^a^	7.53 ± 0.82^a^	7.63 ± 1.90^a^	7.25 ± 0.70^a^
Texture	7.04 ± 0.89^a^	7.10 ± 0.94^a^	6.87 ± 1.02^a^	7.68 ± 0.84^a^	6.84 ± 2.10^a^
Overall acceptability	7.24 ± 1.40^a^	7.02 ± 1.51^a^	7.13 ± 0.95^a^	7.71 ± 1.79^a^	7.28 ± 1.84^a^

Mean values ± standard deviation with different small letters in the same row have significance different (*p* < 0.05).
